# Antimicrobial resistance among bacterial pathogens of public health interest in Vietnam from a One Health perspective: protocol for a systematic review and meta-analysis

**DOI:** 10.1136/bmjopen-2025-105949

**Published:** 2026-05-03

**Authors:** Max van Wijk, Soe Yu Naing, Trang T Vu, Hai H T Ngo, Ha T Le, Thao P Nguyen, Bich N T Vu, Thomas Kesteman, H Rogier van Doorn, Jaap A Wagenaar, Sonia Lewycka

**Affiliations:** 1 Oxford University Clinical Research Unit Vietnam, Hanoi, Vietnam; 2 Division of Infectious Diseases and Immunology, Utrecht University Faculty of Veterinary Medicine, Utrecht, the Netherlands; 3 Centre for Tropical Medicine and Global Health, University of Oxford Nuffield Department of Medicine, Oxford, UK

**Keywords:** Systematic Review, INFECTIOUS DISEASES, Anti-Bacterial Agents, Epidemiology

## Abstract

**Abstract:**

**Introduction:**

Antimicrobial resistance (AMR) is a pressing public health threat, and the prevalence of AMR is particularly high in Vietnam. A comprehensive review of AMR in humans, animals and/or the environment in Vietnam has however not been conducted to date. This systematic review aims to address this evidence gap and will collect and aggregate findings from literature on AMR in Vietnam among bacterial pathogens of public health interest from a One Health perspective. The results from this countrywide literature review may serve as a guiding tool for policymakers, medical practitioners, veterinarians and other relevant stakeholders in Vietnam and outside the country. This review will also identify specific areas where critical information is lacking, which will be of value for future surveillance programmes and epidemiological research.

**Methods and analysis:**

Studies reporting primary data on antimicrobial susceptibility testing from human (both community and hospital settings), animal and environmental samples in Vietnam will be included. The search will be conducted in PubMed, Web of Science, Embase and Scopus. In addition, Google Scholar will be used to retrieve literature published in Vietnamese and Open Access Theses and Dissertations will be used to seek relevant PhD dissertations. More than 18 different pathogens will be included in this review, mainly based on the 2017 and 2024 WHO bacterial priority pathogens list and the WHO Global Antimicrobial Resistance and Use Surveillance System. Risk of bias (quality) assessment of included studies will be conducted using (1) The thirteen mandatory elements of the Microbiology Investigation Criteria for Reporting Objectively checklist and (2) The bias appraisal framework of Hoy *et al*. The outcome of this literature review will be the prevalence of resistance among selected bacteria, stratified by setting (human (hospital or community), animal and the environment). Pooled prevalence estimates will be calculated for each of the selected antimicrobial-bacterial pathogen combinations. This literature review will be reported in accordance with the Preferred Reporting Items for Systematic Reviews and Meta-Analyses guidelines.

**Ethics and dissemination:**

Ethical approval is not required as no primary data are to be collected. The results from this review will be submitted for publication in a peer-reviewed journal.

**PROSPERO registration number:**

PROSPERO CRD420251047399.

**STRENGTHS AND LIMITATIONS OF THIS STUDY**

- PubMed, Embase, Web of Science, Scopus, Google Scholar and Open Access Theses and Dissertations will be searched for relevant records, ranging from peer-reviewed publications to PhD dissertations in both English and Vietnamese languages to mitigate both publication and language bias.
- This literature review will be reported in accordance with the PRISMA-2020 guidelines and is prospectively registered in the PROSPERO database.
- The Microbiology Investigation Criteria for Reporting Objectively (MICRO) checklist and bias appraisal framework by Hoy *et al* will be used for the quality appraisal of selected articles. No assessment of meta-biases will be performed. A Grading of Recommendations, Assessment, Development and Evaluations (GRADE) assessment will be performed for each outcome.
- Bacterial pathogens to be included in the current review were carefully selected with the aim of generating a comprehensive overview of the trends and current status of antimicrobial resistance in Vietnam from a One Health perspective, but the outcomes of this review may be limited in their generalisability to other pathogens.

## Introduction

 Antimicrobial resistance (AMR) is a pressing public health threat. In 2021, a total of 1.1 million deaths were attributed to AMR globally, with predictions indicating that AMR will become the leading cause of death by 2050.^1 2^ Southeast Asia, and Vietnam in particular, is identified as a hotspot for the emergence and spread of AMR,^3 4^ but there is a lack of systematic data collection in the region.^5^

A systematic review from 2019 on carbapenem and colistin resistance among Enterobacterales in eleven countries in Southeast Asia concluded that Vietnam had the highest prevalence of carbapenem resistance among *Klebsiella pneumoniae*.^6^ National surveillance data from 2016 to 2017 from thirteen hospitals in Vietnam also demonstrated high proportions of resistance among various bacterial pathogens, with a substantial increase in AMR prevalence compared with 2012–2013.^7^ The proportion of methicillin-resistant and multi-drug resistant (MDR) *Staphylococcus aureus* among clinical isolates from a general hospital in Hanoi between 2014–2021 was found to be as high as 73% and 60%, respectively.^8^ Results from a cross-sectional study from 2018 to 2019 demonstrated that 94% of the general population in northern Vietnam carried third-generation cephalosporin-resistant Enterobacterales.^9^ The observed patterns of AMR in Vietnam are not limited to humans but are also seen in animals and the environment. A study from 2022 on AMR among *Escherichia coli* in southern Vietnam found that 63% of all isolates from chickens were MDR.^10^ Findings from a pilot surveillance programme from 2017 to 2019 in five provinces in Vietnam demonstrated that nearly all *E. coli* and non-typhoidal *Salmonella* isolates (respectively 94% and 89%) obtained from chicken and pigs at slaughterhouses were MDR.^11^ The high proportions of AMR were similar to a study from 2015 in which 87% of pig isolates and 70% of chicken isolates were considered MDR.^12^ Similar findings are observed in aquaculture, with Vietnam being one of the countries in Asia with the highest AMR rates in the marine environment.^13 14^

The prevalence of AMR is particularly high in Vietnam, a result of the long-term inappropriate and unregulated use of antimicrobials in humans, animals and plants.^4 5^ Based on the findings from qualitative research in northern Vietnam in 2020, it was hypothesised that the misuse of antimicrobials in communities is due to an inadequate healthcare system and unequal access to quality healthcare.^15^ The imprudent use of antimicrobials is not limited to the human sector. Vietnam is one of the countries in Asia with the highest antimicrobial consumption in aquaculture.^16^ Survey-based research from 2015 found that the majority (72%) of freshwater fish or shrimp farms reported using antimicrobials.^17^ Another analysis on the use of commercial chicken and pig feed products from the same year found that more than half (57%) of feed products were mixed with antimicrobials, including amoxicillin, colistin and tetracyclines.^18^ The high usage of critical antimicrobials in animals may also have a spillover effect on humans and the environment, which underlines the need for One Health solutions to mitigate AMR.

Most AMR surveillance systems in Vietnam rely on clinical isolates from humans, but less is known about the prevalence of AMR among the general population, animals and the environment. Initiatives such as WHO’s Global Antimicrobial Resistance and Use Surveillance System (GLASS) and the Food and Agriculture Organisation of the United Nations’ InFARM aim to systematically collect data on AMR in humans and animals, respectively, although they rely on self-reported data from participating countries. Published systematic reviews on AMR in Vietnam and Southeast Asia typically focus on specific bacterial species,^19^,^21^ but do not provide a comprehensive overview of the published evidence. Recently, Gach *et al* conducted a systematic literature review on the prevalence of AMR in humans in Indonesia.^22^ A comprehensive review on AMR in humans, animals and/or the environment in Vietnam has, however, not been conducted to date.

The objective of this systematic literature review and meta-analysis is to collect and aggregate all literature on patterns of AMR in bacterial pathogens of public health importance in Vietnam from a One Health perspective. Specifically, this literature review will summarise the prevalence of AMR among pathogens of public health interest, primarily based on the WHO 2017 and 2024 bacterial priority pathogen lists (BPPL) and GLASS-AMR manual, which represent resistant bacteria with the greatest unmet need and the most significant public health burden.^23^,^25^ This countrywide study can be a guiding tool for policymakers, medical practitioners and other relevant stakeholders in Vietnam and outside the country. This review will also identify specific areas where critical information is lacking.

## Methods and analysis

This protocol is written in accordance with the Preferred Reporting Items in Systematic Review and Meta-Analysis (PRISMA) for Systematic Review Protocol standards (PRISMA-P) (see online supplemental file 1 for the completed PRISMA-P checklist). This systematic review will be reported in accordance with the PRISMA guidelines and the PRISMA 2020 checklist. The protocol has been registered in the International Prospective Register of Systematic Reviews (PROSPERO) database (CRD420251047399). The initial literature search was conducted in July 2025, and the systematic review is planned to be completed by July 2026.

### Study overview

The primary outcome of interest in this systematic review is the prevalence of AMR in Vietnam among bacteria of public health interest. The primary outcome of interest in this systematic review is the prevalence of AMR in Vietnam among selected bacterial pathogens: *Acinetobacter* spp.*, Aeromonas* spp., *Campylobacter* spp., Enterobacterales (including *Klebsiella pneumoniae, Escherichia coli, Enterobacter* spp.), *Enterococcus* spp.*, Haemophilus influenzae, Helicobacter pylori, Neisseria gonorrhoeae*, *Neisseria meningitidis, Pseudomonas aeruginosa, Salmonella* spp.*, Shigella* spp.*, Staphylococcus aureus, Streptococcus pneumoniae, Streptococcus suis,* Group A Streptococci, Group B Streptococci and *Vibrio* spp. All bacterial pathogens from the 2017 and 2024 WHO BPPL and the GLASS-AMR manual are included. In addition, *Aeromonas* spp., *Streptococcus suis, Enterococcus* spp. (instead of *Enterococcus faecium*) and *Vibrio* spp. will be included. *Mycobacterium tuberculosis* will not be included due to their distinct diagnostic features. Please see online supplemental file 2 for an overview of pathogens to be included in this review.

### Eligibility criteria

Original articles, in English or Vietnamese language, that include quantitative findings on AMR of at least one of the selected pathogens obtained from human, animal or environmental samples in Vietnam will be included. In addition, PhD dissertations will be included to collect relevant findings that are not (yet) published. Interventions and comparators (in accordance with the PICO (Population, Intervention, Control, Outcome) framework) are not applicable given the nature of this literature review. The primary outcome of this study is the prevalence of AMR among selected bacterial pathogens stratified per setting (human (community or hospital), animal or the environment) and per antimicrobial. No restrictions to antimicrobials will be applied and findings for every antimicrobial from the original records will be reported and used for further analyses.

Records other than original articles will be excluded. It is expected that the vast majority of included records are findings from observational studies, such as cross-sectional or retrospective studies. Findings from other study types, such as clinical trials, may be included if they provide baseline data on the prevalence of AMR before the population or environment was exposed to the intervention (or control) of interest. Results from trials with a control that is reflective of the current situation will also be included. The outcome of interest is the prevalence of AMR among selected bacteria. No restrictions will be applied to the type of antimicrobial susceptibility testing (AST) or criteria used to interpret isolates as resistant.

The following inclusion criteria will be used:

- Publication type: peer-reviewed manuscripts (English language), published manuscripts (Vietnamese language) or PhD dissertations.
- Language: English or Vietnamese.
- Location: Vietnam.
- Scope: findings on one of the selected bacteria.
- Scope: findings on AST.
- Scope: at least 10 isolates of at least one bacterium of interest.
- Scope: data collection completed after 1 January 2000.

The following exclusion criteria will be used:

- Publication type: other than described above such as review, editorial or conference abstract.
- Data: study has missing numerators/denominators.
- Data: data from Vietnam and outside Vietnam could not be separated.

### Search strategy

Literature will be searched to identify eligible studies published after 1 January 2000. An update of this systematic literature review may be performed before the final analysis to include additional studies that will be published after the initial search date.

The following four databases will be used to retrieve peer-reviewed literature: PubMed, Embase, Web of Science and Scopus. In addition, Google Scholar will be used to retrieve Vietnamese literature and Open Access Theses and Dissertations will be used to collect relevant PhD theses. The search strategy will be developed using a combination of the following concepts:

1. AMR: “antimicrobial resistance”, AMR, “antibiotic resistance”, “drug resistance”, resistance, susceptibility. The following search terms will be used to identify relevant literature published in Vietnamese: “kháng kháng sinh”, “kháng thuốc”.
2. Vietnam (limited to title or abstract).
3. Selected pathogens: *Acinetobacter*, *Aeromonas*, *Campylobacter, Enterobacter,* Enterobacterales, *Enterobacteriaceae, Klebsiella, Escherichia, Enterococcus,* Enterococci*, Hemophilus, Haemophilus, Helicobacter, Neisseria, Pseudomonas, Salmonella, Shigella*, *Staphylococcus*, Streptococci, *Streptococcus*, *Vibrio*.

The search terms within each concept will be using the Boolean operator “OR” and the concepts will be combined using the operator “AND”. Online supplemental file 3 presents a draft search strategy for all databases. The search for Vietnamese literature may be limited to published articles in selected, peer-reviewed journals. The full search strategy for each database will be provided in an appendix to ensure reproducibility.

#### Article screening and selection

All duplicates from the studies retrieved during the searches will be removed before abstract screening using Rayyan, an AI-assisted review tool. Titles and abstracts of all unique records will be screened independently by two reviewers (MW and SYN) for inclusion against the predefined eligibility criteria. Literature written in Vietnamese will be reviewed by native speakers (TTV, HHTN and HTL). Full reports will be obtained for all the records included after the initial screening phase and will again be screened independently by two reviewers to determine which reports meet the in- and exclusion criteria. At this stage, the exclusion reason for each individual record will be recorded, using the hierarchy according to the above-mentioned exclusion criteria. With respect to both abstract and full-text screening, a third reviewer may be asked to independently screen records to resolve potential discrepancies. A PRISMA flow diagram will be added to the final manuscript to summarise the study selection process.

### Risk of bias assessment of individual studies

The (1) Microbiology Investigation Criteria for Reporting Objectively (MICRO) checklist and (2) The bias appraisal framework developed by Hoy *et al* will be used to assess the risk of bias (quality) for each study.^26 27^

The MICRO checklist is a 20-item checklist which defines items to be included in reports of studies involving human clinical microbiology data, including study design, setting, laboratory work, quality assurance, bias and reporting of results.^27^ The thirteen mandatory items from the MICRO checklist will be recorded for each study included in this systematic literature review.

The framework developed by Hoy *et al* is a 10-item checklist which assesses both external validity and internal validity of prevalence studies across four domains (selection bias, nonresponse bias, measurement bias and bias related to the analysis).^26^ Each item will be rated as having a low or high risk of bias, and an overall score will classify studies as low (up to two points), moderate (3 through six points) or high (seven or more points) risk of bias. This framework has also been used to evaluate the risk of bias in a recently published systematic review and meta-analysis on the prevalence of third-generation cephalosporin resistance in Sub-Saharan Africa.^28^

The risk of bias assessment will be conducted independently by two reviewers, with any discrepancies being resolved by a third reviewer. Studies will not be excluded following the assessment according to the MICRO checklist and/or the bias appraisal framework by Hoy *et al*. Sensitivity analyses informed by the outcomes of the risk of bias assessments will be conducted to understand the impact of the quality of included evidence on the overall pooled prevalence estimates.

### Data synthesis and meta-analysis

Summary tables describing the study characteristics, results and results from the risk of bias assessments will be developed. Two researchers will independently extract data from included reports into predefined summary tables. Any disagreements will be resolved through an additional check by a third researcher.

The following data will be extracted from each study:

- Publication details: first author and year of publication.
- Study characteristics: study design (retrospective, cross-sectional or prospective), setting (human, animal or the environment), year(s) of data collection, geographical location, specimen type (eg, blood, rectal swab, urine, dust, water, soil, etc) and organisms included.
  - An additional number of variables will be extracted for studies on human samples, namely: health status (eg, general population or selected disease), age group (children <18 years of age or adults ≥18 years of age), sex (male or female) and healthcare setting (inpatient, outpatient and community).
  - For animal samples: species, setting (healthy, sick).
  - For environmental samples: location (eg, farm, hospital).
- AST results (for each of the selected bacterial pathogens): phenotypic methods used (eg, disc diffusion, etc), interpretative criteria used including the version/year of the respective criterion (eg, Clinical and Laboratory Standards Institute, European Committee on AST, etc), number of isolates analysed, number of isolates with resistance.
- Other results (if available): MDR (yes/no, and definition of MDR), molecular confirmation (yes/no).

Studies in primary care and outpatient clinics will be classified as outpatient setting. Studies for which it is unspecified whether inpatients or outpatients or mixed study populations are included will be classified as hospital setting. If possible, hospital-acquired and community-acquired data will be extracted separately for studies in the inpatient setting. If studies report findings from several settings, timepoints or subgroups, each will be extracted separately. Studies reporting results from working professionals (eg, healthcare professionals, farmers, slaughterhouse workers) will be included in the community analysis. Subgroup analyses will be performed excluding working professionals to understand whether certain professions are at increased risk of carrying resistant pathogens.

It is expected that most studies will not report the minimal inhibitory concentrations for antimicrobials. If available, cut-offs used to categorise isolates as susceptible, intermediate or resistant will be extracted. The interpretations from the authors will therefore be used to determine the prevalence of susceptible, intermediate and/or resistant isolates.

#### Heterogeneity assessment

A heterogeneity assessment of studies selected for the meta-analysis will be performed using the inverse variance index (I² statistic), between-study variance (τ²), and Cochran’s Q test. An index above 75% and a p value below 0.05 will be considered as significant heterogeneity. Heterogeneity will further be assessed through prespecified subgroup analyses (see *subgroup analysis*). Additional subgroup (sensitivity) analyses may be explored depending on the number of included studies and/or heterogeneity of included studies. If considerable heterogeneity is observed, it will be noted that results should be interpreted carefully.

#### Meta-analysis

Pooled prevalence estimates for each antimicrobial-bacterial pathogen combination will be calculated using a random-effects binomial generalised mixed model, in which the number of resistant isolates is modelled as a binomial outcome with the total number of tested isolates as the denominator, and between-study heterogeneity is accounted for through a random study effect. The pooled prevalence will be calculated separately for the community setting, hospital setting, animals and the environment. A 95% CI around the point estimate and a forest plot will be created for each antimicrobial-bacterial pathogen combination and setting for which the number of studies is more than one.

#### Subgroup analyses

The following subgroup analyses will be performed:

- Period of data collection: 2000–2004, 2005–2009, 2010–2014, 2015–2019 and 2020–2024.
- Location:
  - North, central, south Vietnam.
  - Urban versus rural.
- Age category (only for studies on human samples): neonates, children <5 years of age, children 5 to 17 years-of-age, children <18 years of age and adults ≥18 years of age.
- Healthcare setting (only for studies on human samples): inpatient, outpatient and community.
- Community subgroups (only for studies on human samples): general community, various working professionals (eg, healthcare professionals, farmers, slaughterhouse workers).
- Animal species (only for studies on animal samples): for example, cattle, small ruminants, pigs (reproduction, fattening), chicken (layer, broiler, native species), ducks, aquaculture (check for different types of animals).
- Type of sample:
  - For example, live animals, samples from dead animals, meat (only for studies on animal samples).
  - For example, blood, rectal swab, urine (only for studies on human and/or animal samples).
  - For example, dust, water (wastewater, surface water and drinking water), soil (only for studies on environmental samples).

Additional subgroup (sensitivity) analyses, such as stratified for AST method, interpretative criteria and sample type, may be explored depending on the number of included studies and/or heterogeneity of included studies.

### Assessment of meta-biases and confidence in cumulative evidence

Given the nature of the current literature review, no assessment of meta-biases will be performed. A Grading of Recommendations, Assessment, Development and Evaluations assessment will be performed for each pooled prevalence estimate (as described, pooled prevalence estimates will be calculated separately for the community setting, hospital setting, animals and the environment).

## Discussion

This systematic literature review and meta-analysis of published literature aims to summarise the prevalence of AMR among common bacterial pathogens in Vietnam from a One Health perspective. To our knowledge, this will be the first systematic literature that collects and aggregates all the available evidence on AMR patterns in Vietnam.

It must be noted that a limited number of studies may be identified for some of the selected antimicrobial-bacterial pathogen combinations, which will reduce the power of our results. There also is a potential for considerable statistical heterogeneity as a result of the various settings in which studies may have taken place. This may also create the potential limitation that not all prespecified subgroup analyses can be performed.

The results from this countrywide literature review may serve as a guiding tool for policy makers, medical practitioners, veterinarians and other relevant stakeholders in Vietnam and outside the country. This review will also identify specific areas where critical information is lacking, which will be of value for future surveillance programmes and epidemiological research. The findings may support the prioritisation of pathogen-antimicrobial combinations for inclusion in regional or national surveillance and stewardship programmes in Vietnam. By providing a comprehensive overview of AMR patterns from a One Health perspective in Vietnam, the outcomes of this systematic review and meta-analysis may also contribute to evidence-based policy making aimed at mitigating the growing burden of AMR in Vietnam.

## Ethics and dissemination

Ethical approval is not required as no primary data are to be collected. All OUCRU studies fall under the responsibility of the Oxford Tropical Research Ethics Committee (OxTREC; oxtrec@admin.ox.ac.uk). The results from this review will be submitted for publication in a peer-reviewed journal. Interim and/or final findings may also be presented in (inter)national conferences. Researchers who contribute to this review will be offered authorship on publication(s) and abstract(s) in accordance with the International Committee of Medical Journal Editors (ICMJE) guidelines.

## Supplementary material

10.1136/bmjopen-2025-105949 online supplemental file 1

10.1136/bmjopen-2025-105949 online supplemental file 2

10.1136/bmjopen-2025-105949 online supplemental file 3
