# Supplementary material for: Antimicrobial resistance among bacterial pathogens of public health interest in Vietnam from a One Health perspective: protocol for a systematic review and meta-analysis
Source: BMJ Open. 2026 May 3;16(4):e105949. doi: 10.1136/bmjopen-2025-105949 (PMC13141021; doi:10.1136/bmjopen-2025-105949)
Supplement: online supplemental file 2 [file bmjopen-16-4-s002.docx]

**Table S1. Overview of pathogens to be included in this systematic review**

| **Pathogen** | **WHO BPPL 2017^1^** | **WHO BPPL 2024^2^** | **WHO GLASS-AMR manual 2021^3^** | **Rationale to justify inclusion**  *For pathogens not listed in the WHO BPPL or GLASS-AMR manual* |
| --- | --- | --- | --- | --- |
| *Acinetobacter* spp. | ✔  *Acinetobacter baumannii*  (carbapenem-resistant) | ✔  *Acinetobacter baumannii*  (carbapenem-resistant) | ✔ |  |
| *Aeromonas* spp. |  |  |  | Highly prevalent pathogen in **aquaculture** and may also cause infection in humans. |
| *Campylobacter* spp. | ✔  (fluoroquinolone-resistant) |  |  |  |
| Enterobacterales | ✔  *Enterobacteriaceae* spp.  (carbapenem-resistant & ESBL-producing) | ✔  (third-generation cephalosporin- & carbapenem-resistant) | *✔*  *E. coli*  *K. pneumonia* |  |
| *Enterococcus* spp. | ✔  *Enterococcus faecium*  (vancomycin-resistant) | ✔  *Enterococcus faecium*  (vancomycin-resistant) |  | *Enterococcus* spp. instead of *Enterococcus faecium* will be considered to ensure inclusion of *Enterococcus faecalis* among others which cause infection in animals (and may also cause infection in humans). |
| *Haemophilus influenzae* | ✔  (ampicillin-resistant) | ✔  (ampicillin-resistant) | ✔ |  |
| *Helicobacter pylori* | ✔  (clarithromycin-resistant) |  |  |  |
| *Neisseria gonorrhoeae* | ✔  (cephalosporin- & fluoroquinolone-resistant) | ✔  (third-generation cephalosporin- & fluoroquinolone-resistant) | ✔ |  |
| *Neisseria meningitidis* |  |  | ✔ |  |
| *Pseudomonas aeruginosa* | ✔  (carbapenem-resistant) | ✔  (carbapenem-resistant) | ✔ |  |
| *Salmonella* spp. | ✔  (fluoroquinolone-resistant) | ✔   - Non-typhoidal *Salmonella*   (fluoroquinolone-resistant)   - *Salmonella* Typhi   (fluoroquinolone-resistant) | ✔   - Non-typhoidal *Salmonella* spp. - *S. enterica* Typhi - *S. enterica* Paratyphi A. |  |
| *Shigella* spp. | ✔  (fluoroquinolone-resistant) | ✔  (fluoroquinolone-resistant) | ✔ |  |
| *Staphylococcus aureus* | ✔  (methicillin- & vancomycin-resistant) | ✔  (methicillin-resistant) | ✔ |  |
| *Staphylococcus pneumoniae* | ✔  (penicillin-non-susceptible) | ✔  (macrolide-resistant) | ✔ |  |
| *Streptococcus suis* |  |  |  | Highly prevalent pathogen in **swine** and may also cause infection in humans. |
| Group A Streptococci |  | ✔  (macrolide-resistant) |  |  |
| Group B Streptococci |  | ✔  (penicillin-resistant) |  |  |
| *Vibrio* spp. |  |  |  | Highly prevalent pathogen in **aquaculture** and may also cause infection in humans. |

Abbreviations: ESBL (extended spectrum beta-lactamases), BPPL (bacterial priority pathogen list), WHO (World Health Organization), spp. (species).

References:

1. Tacconelli E, Carrara E, Savoldi A, Harbarth S, Mendelson M, Monnet DL, et al. Discovery, research, and development of new antibiotics: the WHO priority list of antibiotic-resistant bacteria and tuberculosis. Lancet Infect Dis. 2018;18(3):318-27.

2. World Health Organization (WHO). WHO Bacterial Priority Pathogens List, 2024: bacterial pathogens of public health importance to guide research, development and strategies to prevent and control antimicrobial resistance. Geneva; 2024.

3. World Health Organization (WHO). Global Antimicrobial Resistance and Use Surveillance System - GLASS-AMR Manual 2.0. 2021.
