# Supplementary material for: Antimicrobial resistance among bacterial pathogens of public health interest in Vietnam from a One Health perspective: protocol for a systematic review and meta-analysis
Source: BMJ Open. 2026 May 3;16(4):e105949. doi: 10.1136/bmjopen-2025-105949 (PMC13141021; doi:10.1136/bmjopen-2025-105949)
Supplement: online supplemental file 3 [file bmjopen-16-4-s003.docx]

**Table 1. Draft of search strategy**

|  | **Search term** | **Number of hits** |
| --- | --- | --- |
| **Category 1: Literature published in English (and Vietnamese)^*^** | | |
| PubMed | (“antimicrobial resistance” OR AMR OR “antibiotic resistance” OR “drug resistance” OR susceptibility OR resistance) AND (Vietnam[tiab] OR “Viet Nam”[tiab]) AND (*Acinetobacter* OR *Aeromonas* OR *Campylobacter* OR *Enterobacter* OR Enterobacterales OR *Enterobacteriaceae* OR *Klebsiella* OR *Escherichia* OR *Enterococcus* OR Enterococci OR *Hemophilus* OR *Haemophilus* OR *Helicobacter* OR *Neisseria* OR *Pseudomonas* OR *Salmonella* OR *Shigella* OR *Staphylococcus* OR Streptococci OR *Streptococcus* OR *Vibrio*)  Filter: from 2000/JAN/1 | **583** |
| Embase | #1 ‘antimicrobial resistance’ OR AMR OR ‘antibiotic resistance’ OR ‘drug resistance’ OR susceptibility OR resistance | 2,226,028 |
|  | #2 vietnam:ab,ti OR ‘viet nam’:ab,ti | 27,639 |
|  | #3 *Acinetobacter* OR *Aeromonas* OR *Campylobacter* OR *Enterobacter* OR Enterobacterales OR *Enterobacteriaceae* OR *Klebsiella* OR *Escherichia* OR *Enterococcus* OR Enterococci OR *Hemophilus* OR *Haemophilus* OR *Helicobacter* OR *Neisseria* OR *Pseudomonas* OR *Salmonella* OR *Shigella* OR *Staphylococcus* OR Streptococci OR *Streptococcus* OR *Vibrio* | 1,535,878 |
|  | #1 AND #2 AND #3 | 746 |
|  | **#1 AND #2 AND #3 AND [01-01-2000]/sd** | **669** |
| Web of Science | #1 “antimicrobial resistance” OR AMR OR “antibiotic resistance” OR “drug resistance” OR susceptibility OR resistance [All fields] | 2,824,787 |
|  | #2 Vietnam OR “Viet Nam” [Abstract] | 44,234 |
|  | #3 *Acinetobacter* OR *Aeromonas* OR *Campylobacter* OR *Enterobacter* OR Enterobacterales OR *Enterobacteriaceae* OR *Klebsiella* OR *Escherichia* OR *Enterococcus* OR Enterococci OR *Hemophilus* OR *Haemophilus* OR *Helicobacter* OR *Neisseria* OR *Pseudomonas* OR *Salmonella* OR *Shigella* OR *Staphylococcus* OR Streptococci OR *Streptococcus* OR *Vibrio* [All fields] | 1,326,113 |
|  | #1 AND #2 AND #3 | 600 |
|  | #1 AND #2 AND #3  Filter: from 2000/JAN/1 | 583 |
|  | #4 Vietnam OR “Viet Nam” [Title] | 37,456 |
|  | #1 AND #2 AND #4 | 408 |
|  | #1 AND #2 AND #4  Filter: from 2000/JAN/1 | 400 |
|  | **Total Web of Science** | **983**  **(583 + 400)** |
| Scopus | #1 “antimicrobial resistance” OR AMR OR “antibiotic resistance” OR “drug resistance” OR susceptibility OR resistance [All fields] | 9,791,014 |
|  | #2 Vietnam OR “Viet Nam” [Article title, Abstract, Keywords] | 91,487 |
|  | #3 *Acinetobacter* OR *Aeromonas* OR *Campylobacter* OR *Enterobacter* OR Enterobacterales OR *Enterobacteriaceae* OR *Klebsiella* OR *Escherichia* OR *Enterococcus* OR Enterococci OR *Hemophilus* OR *Haemophilus* OR *Helicobacter* OR *Neisseria* OR *Pseudomonas* OR *Salmonella* OR *Shigella* OR *Staphylococcus* OR Streptococci OR *Streptococcus* OR *Vibrio* [All fields] | 3,608,840 |
|  | #1 AND #2 AND #3 | 1,880 |
|  | **#1 AND #2 AND #3**  **Filter: from 2000** | **1,785** |
| **Category 2: Literature published in Vietnamese^**^** | | |
| Google Scholar | #1 "kháng kháng sinh" OR "kháng thuốc" | 5,900 |
|  | #2 *Acinetobacter* OR *Aeromonas* OR *Campylobacter* OR *Enterobacter* OR Enterobacterales OR *Enterobacteriaceae* OR *Klebsiella* OR *Escherichia* OR *Enterococcus* OR Enterococci OR *Hemophilus* OR *Haemophilus* OR *Helicobacter* OR *Neisseria* OR *Pseudomonas* OR *Salmonella* OR *Shigella* OR *Staphylococcus* OR Streptococci OR *Streptococcus* OR *Vibrio* | 9,580,000 |
|  | ("kháng kháng sinh" OR "kháng thuốc") AND (*Acinetobacter* OR *Aeromonas* OR *Campylobacter* OR *Enterobacter* OR Enterobacterales OR *Enterobacteriaceae* OR *Klebsiella* OR *Escherichia* OR *Enterococcus* OR Enterococci OR *Hemophilus* OR *Haemophilus* OR *Helicobacter* OR *Neisseria* OR *Pseudomonas* OR *Salmonella* OR *Shigella* OR *Staphylococcus* OR Streptococci OR *Streptococcus* OR *Vibrio*) | 3,280 |
|  | ("kháng kháng sinh" OR "kháng thuốc") AND (*Acinetobacter* OR *Aeromonas* OR *Campylobacter* OR *Enterobacter* OR Enterobacterales OR *Enterobacteriaceae* OR *Klebsiella* OR *Escherichia* OR *Enterococcus* OR Enterococci OR *Hemophilus* OR *Haemophilus* OR *Helicobacter* OR *Neisseria* OR *Pseudomonas* OR *Salmonella* OR *Shigella* OR *Staphylococcus* OR Streptococci OR *Streptococcus* OR *Vibrio*)  Filter: From 2000 | **3,150** |
| **Category 3: PhD dissertations^**^** | | |
| OATD | #1 (“antimicrobial resistance”) OR (“AMR”) OR (“antibiotic resistance”) OR (“drug resistance”) OR (“susceptibility”) OR (“resistance”) | 328,870 |
|  | #2 (“Vietnam”) OR (“Viet Nam”) | 14,258 |
|  | #3 (“*Acinetobacter”*) OR (“*Aeromonas”*) (“*Campylobacter”*) OR (“*Enterobacter”*) OR (“Enterobacterales*”*) OR (“*Enterococcus”*) OR (“*Escherichia”*) OR (“*Haemophilus”*) OR (“*Helicobacter”*) OR (“*Klebsiella”*) OR (“*Morganella”*) OR (“*Neisseria”*) OR (“*Proteus”*) OR (“*Providencia”*) OR (“*Pseudomonas”*) OR (“*Salmonella”*) OR (“*Serratia”*) OR (“*Shigella”*) OR (“*Staphylococcus”*) OR (“Streptococci*”*) OR (“*Streptococcus”*) OR (“*Vibrio*”) | 72,563 |
|  | **#1 AND #2 AND #3** | **63** |

Results based on a search on (*) 28 July 2025 and (**) 24 February 2026.
