# Supplementary material for: Antimicrobial resistance among bacterial pathogens of public health interest in Vietnam from a One Health perspective: protocol for a systematic review and meta-analysis
Source: BMJ Open. 2026 May 3;16(4):e105949. doi: 10.1136/bmjopen-2025-105949 (PMC13141021; doi:10.1136/bmjopen-2025-105949)
Supplement: Supplementary file 4 [file bmjopen-2025-105949.reviewer_comments.pdf]

PEER REVIEW HISTORY

BMJ Open publishes all reviews undertaken for accepted manuscripts. Reviewers are asked to complete a checklist review form and are provided with free text boxes to elaborate on their assessment. These free text comments are reproduced below.

ARTICLE DETAILS

Title (Provisional)

Antimicrobial resistance among bacterial pathogens of public health interest in Vietnam from a One Health perspective: protocol for a systematic review and meta-analysis

Authors

van Wijk, Max; Naing, Soe Yu; Vu, Trang T.; Ngo, Hai H.T.; Le, Ha T.; Nguyen, Thao P.; Vu, Bich N.T.; Kesteman, Thomas; van Doorn, H. Rogier; Wagenaar, Jaap A; Lewycka, Sonia

---

VERSION 1 - REVIEW

**Reviewer**	**1**
**Name**	**Thomrongsuwannakij, Thotsapol**
**Affiliation**	**Walailak University**
**Date**	**08-Aug-2025**
**COI**	**None**

General Comments:

The topic is timely and highly relevant, focusing on antimicrobial resistance (AMR) in Vietnam from a One Health perspective. The proposed systematic review and meta-analysis address an important public health concern. The methodology is clearly described and appears to be rigorous and reproducible.

Major Comments:

Lack of Results in the Abstract:

The abstract does not include any preliminary or expected results. Although this is a protocol paper, it would be helpful to briefly outline the anticipated outcomes or key variables of interest.

Absence of Results Section:

As this is a protocol, it is understood that results are not yet available. However, it would be beneficial to explicitly state this in the manuscript to avoid confusion.

Limited Discussion:

The discussion section is relatively brief. Expanding it to include more detail on the potential implications of the review findings and how they might inform policy or practice in Vietnam would strengthen the manuscript.

**Reviewer**	**2**
**Name**	**khan, maria**
**Affiliation**	**Rehman Medical Institute**
**Date**	**28-Oct-2025**
**COI**	**None**

....

**Reviewer**	**3**
**Name**	**Bakon, Sophia**
**Affiliation**	**National Institutes of Health, Institute for Medical Research**
**Date**	**08-Dec-2025**
**COI**	**None**

The protocol for a systematic review follows a very clear flow. Comprehensible by the readers.

**Reviewer**	**4**
**Name**	**Chang, Jie**
**Affiliation**	**Xi'an Jiaotong University, Department of Pharmacy Administration and Clinical Pharmacy, School of Pharmacy**
**Date**	**04-Feb-2026**
**COI**	**None**

The topic is highly relevant given Vietnam’s documented high burden of AMR across human, animal, and environmental sectors. The rationale is clearly articulated; overall, this protocol is of good quality. Below are a few points I raised.

First, the manuscript states that pathogens are selected based on the WHO 2017 and 2024 bacterial priority pathogen lists and GLASS-AMR, with some additional inclusions. It would be helpful to provide a brief justification for these deviations based on Vietnam context relevance.

Also, pls clarify how consistency across the two WHO lists will be handled analytically.

Given the anticipated substantial heterogeneity across study designs, settings, populations, laboratory methods, and interpretive criteria, the feasibility of pooled prevalence estimates for all pathogen–antimicrobial combinations may be limited.

The authors may wish to further specify ,first, criteria for deciding when meta-analysis will not be performed and results will instead be narratively synthesized, and second whether transformations will be used to stabilise variances in prevalence meta-analyses.

Third, the protocol points out that no restrictions will be applied to AST methods or interpretive criteria and that authors' interpretations will be used. While understandable, this approach may introduce systematic bias over time and across settings. The authors should clarify whether sensitivity analyses will be conducted to assess robustness.

Forth, the strengths and limitations section states that a GRADE assessment will be performed for each outcome, but the Methods section does not describe how GRADE will be applied to prevalence estimates derived largely from observational studies. Additional clarification on how certainty of evidence will be rated would improve methodological transparency.

VERSION 1 - AUTHOR RESPONSE

Reviewer	Comment	Response
Reviewer 1	The abstract does not include any preliminary or expected results. Although this is a protocol paper, it would be helpful to briefly outline the anticipated outcomes or key variables of interest.<br>****Comment from the Editor: Please do not include a Results section, as this is not part of formatting requirements for Protocol articles.*	Anticipated outcomes and variables of interest that will be collected are described in the "data synthesis and meta-analysis" subsection of the methodology section. We have therefore not made any changes to the manuscript based on this comment.
	As this is a protocol, it is understood that results are not yet available. However, it would be beneficial to explicitly state this in the manuscript to avoid confusion.<br>****Comment from the Editor: Please do not include a Results section, as this is not part of formatting requirements for Protocol articles.*	In line with the comment from the Editor: we have not made any changes to the manuscript based on this comment.
	The discussion section is relatively brief. Expanding it to include more detail on the potential implications of the review findings and how they might inform policy or practice in Vietnam would strengthen the manuscript.	Thank you for the suggestion. We have added two sentences to clarify how the findings from this review may inform and/or contribute to policy and practice (surveillance and stewardship programs) in Vietnam (see lines 309-312: "*The findings may support the prioritisation of pathogen-antimicrobial combinations for inclusion in regional or national surveillance and stewardship programs in Vietnam. By providing a comprehensive overview of*

		AMR patterns from a One Health perspective in Vietnam, the outcomes of this systematic review and meta-analysis may also contribute to evidence-based policy making aimed at mitigating the growing burden of AMR in Vietnam.”).
Reviewer 2	No comments	We thank the reviewer for their time to read and assess the manuscript. No changes were made to the manuscript based on the feedback from reviewer 2.
Reviewer 3	No comments	We thank the reviewer for their time to read and assess the manuscript. No changes were made to the manuscript based on the feedback from reviewer 3.
Reviewer 4	First, the manuscript states that pathogens are selected based on the WHO 2017 and 2024 bacterial priority pathogen lists and GLASS-AMR, with some additional inclusions. It would be helpful to provide a brief justification for these deviations based on Vietnam context relevance.<br>Also, pls clarify how consistency across the two WHO lists will be handled analytically.	Thank you for the suggestion. We have added an overview (Supplementary file 2) with the pathogens to be included in this systematic review. This overview includes an overview of pathogens that are included in the WHO 2017 and 2014 BPPL and GLASS-AMR manual. A rationale to justify the additional inclusions (*Aeromonas* spp., *Streptococcus suis*, and *Vibrio* spp.) has been provided.<br><br>Specifically:<br>- *Aeromonas* spp. and *Vibrio* spp. have been added as it is a highly prevalent pathogen in aquaculture and may also cause infection in humans.<br>- *Streptococcus suis* has been added as it is a highly prevalent pathogen in swine and may also cause infection in humans.<br>- *Enterococcus* spp. instead of *Enterococcus faecium* will be considered to ensure inclusion of *Enterococcus faecalis* among others which cause infection in animals (and may also cause infection in humans).
	Given the anticipated substantial heterogeneity across study designs, settings, populations, laboratory methods, and interpretive criteria, the feasibility of pooled prevalence estimates for all pathogen–antimicrobial combinations may be limited.<br>The authors may wish to further specify ,first, criteria for deciding when meta-analysis will not be performed and results will instead be narratively synthesized, and second whether transformations will be used to stabilise variances in prevalence meta-analyses.	Meta analyses will be performed for each pathogen-antimicrobial combination for which two or more studies will be included. Pooled prevalence estimates will be calculated separately for the community setting, hospital setting, animals, and the environment. Heterogeneity assessments, prespecified subgroup analyses, and additional subgroup (sensitivity) analyses will be performed. We will describe which results need to be interpreted carefully, based on the outcomes of these heterogeneity and/or subgroup (sensitivity) analyses (see lines 256-259: “*Additional*

		subgroup (sensitivity) analyses may be explored depending on the number of included studies and/or heterogeneity of included studies. If considerable heterogeneity is observed, it will be mentioned that results need to be interpreted carefully.")<br><br>We will apply a binomial generalized mixed model to calculate prevalence estimates. This has been added to the manuscript (see lines262-265: "*Pooled prevalence estimates for each antimicrobial-bacterial pathogen combination will be calculated using a random-effects binomial generalized mixed model, in which the number of resistant isolates is modelled as a binomial outcome with the total number of tested isolates as the denominator, and between-study heterogeneity is accounted for through a random study effect*").
	Third, the protocol points out that no restrictions will be applied to AST methods or interpretive criteria and that authors' interpretations will be used. While understandable, this approach may introduce systematic bias over time and across settings. The authors should clarify whether sensitivity analyses will be conducted to assess robustness.	We agree and are aware of the biases that may be introduced by including all studies that meet the inclusion criteria, regardless of AST method or interpretative used. As described in the manuscript, various scenario (sensitivity) analyses will be conducted to address and assess robustness of the pooled prevalence estimates (see the "subgroup analyses" subsection in lines 188-290: "*Additional subgroup (sensitivity) analyses, such as stratified for AST method, interpretative criteria, and sample type, may be explored depending on the number of included studies and/or heterogeneity of included studies.*").
	Forth, the strengths and limitations section states that a GRADE assessment will be performed for each outcome, but the Methods section does not describe how GRADE will be applied to prevalence estimates derived largely from observational studies. Additional clarification on how certainty of evidence will be rated would improve methodological transparency.	A GRADE assessment will be performed for each pooled prevalence estimate (as described in the Methods section, pooled prevalence estimates will be calculated separately for the community setting, hospital setting, animals, and the environment). The overall quality, and specifically the risk of bias, will be assessed for each individual study through the MICRO checklist and the bias appraisal framework previously developed by Hoy *et al.* We believe that these three instruments should suffice in assessing the certainty of evidence. In addition, sensitivity analyses informed by the outcomes of the risk of bias assessments will be conducted to understand the impact of the quality of evidence on the overall pooled prevalence estimates of the current review. This has been clarified in the Methods section (see lines 293-296: "*Given the nature of the current literature review, no assessment of*

		meta-biases will be performed. A Grading of Recommendations, Assessment, Development and Evaluations (GRADE) assessment will be performed for each *pooled prevalence estimate (as described, pooled prevalence estimates will be calculated separately for the community setting, hospital setting, animals, and the environment). outcome*.”).

Additional changes made to the manuscript compared to the initial submission

- Following a recently published systematic review and meta-analysis on the prevalence of third-generation cephalosporin resistance in Sub-Saharan Africa[1], we have decided to appraise the risk of bias by using the appraisal framework of Hoy *et al.* (2012)[2] instead of the JBI critical appraisal checklist for studies reporting prevalence.
- We decided to add *Aeromonas* spp. and *Vibrio* spp. as two additional pathogens of interest, in consultation with a key opinion leader on AMR in aquaculture. These two pathogens are highly prevalent in aquaculture and have been added to cover the (aquatic) environment within the One Health scope of this review. A justification from these additions to the pathogens listed in the WHO BPPL and GLASS-AMR manual are described in Supplementary file 2.
- Given the large number of records retrieved through Google Scholar to identify relevant publication in Vietnamese language and Vietnamese journals, we will limit this search to selected, peer-reviewed journals. Journals will be selected in consultation with local researchers. The full search strategy will be provided in the final manuscript to ensure reproducibility and transparency of the search strategy.

References

1. Guido G, Frallonardo L, Asaduzzaman M, Farkas FB, De Vita E, Seni A, et al. Third-generation Cephalosporin resistance in Sub-Saharan Africa: a systematic review and meta-analysis. Commun Med (Lond). 2025;6(1):10.
2. Hoy D, Brooks P, Woolf A, Blyth F, March L, Bain C, et al. Assessing risk of bias in prevalence studies: modification of an existing tool and evidence of interrater agreement. J Clin Epidemiol. 2012;65(9):934-9.

VERSION 2 - REVIEW

Reviewer **4**

Name **Chang, Jie**

Affiliation **Xi'an Jiaotong University, Department of Pharmacy Administration and Clinical Pharmacy, School of Pharmacy**

Date **19-Mar-2026**

COI

My previous comments have been adequately addressed or clearly explained, and I have no further remarks.
